# Supplementary material for: Detailed molecular and epigenetic characterization of the pig IPEC-J2 and chicken SL-29 cell lines
Source: iScience. 2023 Feb 20;26(3):106252. doi: 10.1016/j.isci.2023.106252 (PMC10018572; doi:10.1016/j.isci.2023.106252)
Supplement: Data S2. Complete homer output for identified motifs in Chicken SL-29, related to Tables 5 and 6 — Homer motif analysis results for histone modifications H3K4me1, H3K4me3, H3K27ac, enhancers, and ATAC-seq of chicken SL-29 cell line. Parameters for possible false positives is as mentioned earlier for S5. [file mmc3.zip › Data_S2/S6/Chicken_SL_29/peak_files_ATAC/homerResults/motif2.similar.html]

motif2

## Information for motif2

A
C
T
G
C
G
T
A
A
C
G
T
A
C
G
T
A
G
C
T
A
C
G
T
A
G
T
C
A
C
T
G
  
Reverse Opposite:  

A
G
T
C
A
C
T
G
G
T
C
A
C
T
G
A
C
G
T
A
C
G
T
A
A
C
G
T
A
G
T
C
  

|  |  |
| --- | --- |
| p-value: | 1e-1220 |
| log p-value: | -2.810e+03 |
| Information Content per bp: | 1.934 |
| Number of Target Sequences with motif | 1550.0 |
| Percentage of Target Sequences with motif | 1.78% |
| Number of Background Sequences with motif | 101.0 |
| Percentage of Background Sequences with motif | 0.12% |
| Average Position of motif in Targets | 150.1 +/- 82.8bp |
| Average Position of motif in Background | 151.2 +/- 69.8bp |
| Strand Bias (log2 ratio + to - strand density) | 0.1 |
| Multiplicity (# of sites on avg that occur together) | 3.01 |
| Motif File: | file (matrix) reverse opposite |

### Similar de novo motifs found

|  |  |  |  |  |  |  |  |
| --- | --- | --- | --- | --- | --- | --- | --- |
| Rank | Match Score | Redundant Motif | P-value | log P-value | % of Targets | % of Background | Motif file |
| 1 | 0.815 | G T A C G A C T G A C T G A T C C T A G G T C A G T C A C T G A T G C A A C G T | 1e-873 | -2010.683175 | 1.69% | 0.18% | motif file (matrix) |
| 2 | 0.958 | A G T C A C T G G T C A C T G A C G T A C G T A A C G T A T G C | 1e-602 | -1386.214568 | 2.25% | 0.52% | motif file (matrix) |
| 3 | 0.860 | A G T C A G T C A C T G C G T A C G T A C G T A C G T A A C G T | 1e-586 | -1351.412381 | 1.21% | 0.14% | motif file (matrix) |
| 4 | 0.733 | A C G T A G T C A C T G C G T A A C T G C G T A C G T A A C G T | 1e-537 | -1238.463359 | 0.87% | 0.07% | motif file (matrix) |
| 5 | 0.920 | A G T C T C A G C T G A C G T A C G T A C G T A A C G T G T A C | 1e-392 | -903.289091 | 2.49% | 0.86% | motif file (matrix) |
| 6 | 0.726 | A C G T A G T C A C T G C T A G C G T A C G T A C G T A A C G T | 1e-330 | -761.708642 | 1.06% | 0.21% | motif file (matrix) |
| 7 | 0.700 | A C T G A C G T C G T A A G C T A G C T A C G T A C G T A G T C A C T G A C T G | 1e-214 | -493.901485 | 0.24% | 0.01% | motif file (matrix) |
| 8 | 0.679 | A G T C A C T G T C G A G T C A C T G A C G T A A C T G T C G A T C G A G T A C | 1e-119 | -275.136170 | 0.36% | 0.07% | motif file (matrix) |
